# Bridging horizons beyond CIRCULATE-Japan: a new paradigm in molecular residual disease detection via whole genome sequencing-based circulating tumor DNA assay

**DOI:** 10.1007/s10147-024-02493-4

**Published:** 2024-03-29

**Authors:** Tadayoshi Hashimoto, Yoshiaki Nakamura, Eiji Oki, Shin Kobayashi, Junichiro Yuda, Taro Shibuki, Hideaki Bando, Takayuki Yoshino

**Affiliations:** 1 Translational Research Support Office, National Cancer Center Hospital East, Kashiwa, Japan; 2 Department of Gastroenterology and Gastrointestinal Oncology, National Cancer Center Hospital East, 6-5-1 Kashiwanoha, Kashiwa, Chiba 277-8577 Japan; 3 Department of Surgery and Science, Graduate School of Medical Sciences, Kyushu University, Fukuoka, Japan; 4 Department of Hepatobiliary and Pancreatic Surgery, National Cancer Center Hospital East, Kashiwa, Japan; 5 Department of Hematology, National Cancer Center Hospital East, Kashiwa, Japan

**Keywords:** Molecular residual disease, Circulating tumor DNA, Whole genome sequencing, CIRCULATE-Japan, SCRUM-Japan, MONSTAR-SCREEN

## Abstract

Circulating tumor DNA (ctDNA) is the fraction of cell-free DNA in patient blood that originates from a tumor. Advances in DNA sequencing technologies and our understanding of the molecular biology of tumors have increased interest in exploiting ctDNA to facilitate detection of molecular residual disease (MRD). Analysis of ctDNA as a promising MRD biomarker of solid malignancies has a central role in precision medicine initiatives exemplified by our CIRCULATE-Japan project involving patients with resectable colorectal cancer. Notably, the project underscores the prognostic significance of the ctDNA status at 4 weeks post-surgery and its correlation to adjuvant therapy efficacy at interim analysis. This substantiates the hypothesis that MRD is a critical prognostic indicator of relapse in patients with colorectal cancer. Despite remarkable advancements, challenges endure, primarily attributable to the exceedingly low ctDNA concentration in peripheral blood, particularly in scenarios involving low tumor shedding and the intrinsic error rates of current sequencing technologies. These complications necessitate more sensitive and sophisticated assays to verify the clinical utility of MRD across all solid tumors. Whole genome sequencing (WGS)-based tumor-informed MRD assays have recently demonstrated the ability to detect ctDNA in the parts-per-million range. This review delineates the current landscape of MRD assays, highlighting WGS-based approaches as the forefront technique in ctDNA analysis. Additionally, it introduces our upcoming endeavor, WGS-based pan-cancer MRD detection via ctDNA, in our forthcoming project, SCRUM-Japan MONSTAR-SCREEN-3.

## Introduction

As a groundbreaking technology, liquid biopsy has fundamentally transformed the landscape of cancer treatment [1–4]. The exploration of various blood-borne molecules for cancer surveillance, particularly identification of somatic mutations in circulating tumor DNA (ctDNA) from the primary tumor in cell-free DNA (cfDNA) in plasma, has proven to be a non-invasive detection method for malignancy [5–7]. ctDNA, which has a notably short half-life in plasma of less than 2 h, is rapidly cleared from the bloodstream post-radical surgical resection in the absence of residual cancer [8]. This transient nature of ctDNA is clinically significant, because it serves as a dynamic biomarker for early detection of multiple cancers, detection of molecular residual disease (MRD), and monitoring therapeutic efficacy across a spectrum of tumors. Various liquid biopsy techniques and platforms, including tumor-informed and tumor-agnostic ctDNA assays, have been developed to detect MRD of all tumor types [9–11].

Our initiative, CIRCULATE-Japan, is at the forefront of personalized medicine through MRD testing of patients with colorectal cancer who undergo surgical intervention [12]. This project encompasses the GALAXY study, integrating a comprehensive clinical data registry with longitudinal ctDNA monitoring, and two randomized phase III trials, VEGA and ALTAIR, using the Signatera™ assay from Natera, Inc., predicated on whole exome sequencing to customize a personalized panel analyzing 16 somatic sites [8, 13]. The initial results of the GALAXY study highlighted the profound association between the ctDNA status post-surgery and recurrence risk, suggesting that post-operative adjuvant chemotherapy effectively reduces recurrence in ctDNA-positive patients [14]. To date, CIRCULATE-Japan has an extensive dataset encompassing more than 5500 patients. To corroborate findings, the ALTAIR trial, focusing on patients with a positive ctDNA status at any point post-surgery, and VEGA trial, assessing patients with a negative ctDNA status at 4 weeks post-surgery, are ongoing. The outcomes of these studies are eagerly awaited and expected to provide crucial insights into the efficacy of ctDNA-based therapeutic strategies.

The CIRCULATE-Japan study, which focused exclusively on colorectal cancer, a relatively high-shedding tumor, raised several pertinent issues when extended to other cancer types [15]. Presumably, ctDNA originates from apoptotic and necrotic neoplastic cells. Thus, its detection sensitivity is closely linked to the tumor burden. Microscopic lesions characterized by diminished cell death yield a paucity of ctDNA, thereby engendering formidable challenges in MRD detection [16, 17]. Moreover, sensitive MRD detection is difficult to achieve for tumors characterized by low ctDNA shedding, such as lung adenocarcinoma, luminal-type breast cancer, and pancreatic cancer [18–20]. Furthermore, metastatic foci, such as solitary lung, peritoneal, and brain metastases, exhibit a propensity for false-negative ctDNA [21, 22]. Additionally, clonal hematopoiesis of indeterminate potential (CHIP) further complicates data interpretation, leading to false-positive ctDNA signals [23, 24]. To surmount these technical hurdles, several studies have increased the sequencing depth, albeit with moderate success [25]. Alternatively, paired tumor–normal whole genome sequencing (WGS) focuses on genomic breadth rather than depth with the ability to capture somatic signals from the entire genome, including non-exonic regions, thereby providing a potential solution to these challenges [26].

In view of this, we introduced a pan-cancer-WGS-based-MRD platform under our forthcoming SCRUM-MONSTAR project, named MONSTAR-SCREEN-3. This platform assesses the efficacy of MRD detection via longitudinal ctDNA monitoring in patients of all cancer types, including hematological malignancies, using the WGS-based ultrasensitive assay Precise MRD developed by Myriad Genetics (Salt Lake City, UT, USA). We want to establish proof-of-concept for MRD detection in applicable cancer types, expand the sample cohort for each cancer type, and conduct ctDNA-based clinical trials, thereby fostering a paradigm shift to an MRD-guided treatment strategy. This review summarizes the evolution of ctDNA analysis of all malignancy types, classifying them as hematological and solid tumors, and the current methodologies for MRD detection using ctDNA, including WGS-based MRD assays. Additionally, it introduces our upcoming project beyond CIRCULATE-Japan and presents the future of ctDNA-driven treatment strategies.

### Trajectory of the development of ctDNA analysis for solid tumors

Despite the remarkable achievements in MRD detection by ctDNA analysis across several cancer types, its efficacy is not uniformly applicable to all cancers. While patients with certain cancers have exhibited notable outcomes in some investigations, ctDNA analysis has also demonstrated limited validity because of low sensitivity for specific cancer types [27–29]. However, the feasibility of ctDNA applications is anticipated to enhance in tandem with advancements in assay performance. Here, we review and summarize the significance of ctDNA analysis in representative studies of various solid tumor types (Table 1).

**Table 1:** Summary of reports that investigated ctDNA to detect MRD of various cancer types

Cancer type	Cancer, stage(s)	*N*^a^	Methodology	Brief summary [hazard ratio (HR), ctDNA positive compared with negative]^b^	References
CRC	Stage I–III	130	Signatera™	Recurrence in 87.5% of patients with ctDNA (+) after treatment, post-operative, post-ACT, and post-definitive therapy HR for RFS = 7.2, 17.5, and 43.5	[8]
	Stage II	230	Safe-SeqS	Recurrence in 79% of patients with ctDNA (+) without CTx versus 9.8% of patients with ctDNA (−) without CTx (HR for RFS = 18), post-CTx HR for RFS = 11	[31]
	Stage III	96	Safe-SeqS	Post-operative HR for RFS = 3.8, Estimated 3-year recurrence-free interval (RFI): ctDNA (+) versus (−) = 77% versus 30%, post-CTx HR for RFI = 6.8	[32]
	Stage I–IV	103	Guardant Reveal™	Sensitivity and specificity of landmark recurrence: 55.6% and 100%, landmark HR for RFS = 11.28	[33]
	Stage I–III	150	ddPCR	Post-operative HR for DFS = 17.56, serial HR for DFS = 11.33, post-ACT HR = 10.02, median lead time = 11.5 (m)	[34]
	Stage II–III	240	Geneseeq Prime™ 425 genes	Post-operative HR for RFS = 10.98, post-ACT HR for RFS = 12.76, post-definitive therapy HR = 32.02, mean lead time = 5.01 (m)	[35]
	Stage II	302	Safe-SeqS	Relative risk of receiving ACT in ctDNA-guided group: HR = 1.82, 2-year RFS = 93.5% in the ctDNA-guided group versus 92.4% in the standard care group	[36]
	Stage II–IV	1039	Signatera™	Post-operative HR = 10.0, ctDNA (+) was the most significant prognostic factor in stage II/III (HR 10.82), postoperative HR for benefit from ACT = 6.59	[14]
GC	Stage I–III	46	Custom panel (1021 genes)	Any post-operative time HR for DFS and OS = 14.78 and 7.664, median lead time of ctDNA detection over RI progression = 6 (m)	[40]
	Stage IB–IVA	20	VariantDx	Pre-operative ctDNA was a biomarker for the pathological response, and ctDNA positivity after surgery indicated significantly short RFS (HR = 21.8)	[42]
	EC/GC, stage I–III	295	Signatera™	HR for RFS at any time point after surgery within the MRD window and during the surveillance period: 23.6, 10.7, and 17.7	[43]
PC	Localized	59	ddPCR	Sensitivity and specificity of ctDNA during follow-up: 90% and 88%, median lead time of ctDNA detection over clinical progression = 84 days	[20]
	Stage II	20	dPCR	ctDNA positivity at diagnosis: 43%, post-operative ctDNA (+) predicted poor outcomes, median lead time of ctDNA detection over RI progression = 6.5 (m)	[44]
	Localized	42	Safe-SeqS	Preoperative HR for RFS = 4.1, post-operative HR for RFS and OS = 5.4 and 4.0, respectively, 13/13 (100%) patients with post-operative ctDNA (+) recurred	[45]
	Stage I–IV	27	Custom panel (1017 genes)	Post-operative HR for DFS = 5.20, positive post-operative ctDNA status was an independent prognostic factor for DFS (HR = 3.60)	[46]
	Locally advanced	27	ddPCR	Post-operative HR for OS = 5.019, post-operative ctDNA and CA19-9 values had a cumulative effect on both RFS (*P* = 0.0066) and OS (*P* = 0.0046)	[47]
NSCLC	Stage I–III	24	Custom panel (16 genes)	Post-operative sensitivity and specificity for recurrence: 92.9% and 90.0%, median lead time of ctDNA detection over clinical and RI progression = 70 days	[18]
	Stage I–III	78	Custom-panel (50 genes)	ctDNA detected at or before clinical relapse in 37 out of 45 patients, median lead time of ctDNA detection over clinical progression = 151 days	[49]
	Stage I–III	40	CAPP-seq (128 genes)	Post-operative ctDNA (+) in 94% of patients with recurrence, median lead time of ctDNA detection over RI progression = 5.2 (m)	[50]
	Stage III–IV	77	cSMART (127 genes)	Pre-operative HR for RFS = 3.812, recurrence in postoperative ctDNA (+) patients: 63.3%, median lead time of ctDNA detection over RI progression = 12.6 (m)	[51]
	Stage IA–IIIB	88	RaDaR™	Landmark HR for RFS = 5.48, preoperative HR for RFS = 3.14, median lead time of ctDNA detection over RI progression = 212.5 days	[52]
	Stage I–III	261	CAPP-Seq	NPV of longitudinal undetectable MRD: 96.8%, PPV of longitudinal detectable MRD: 89.1%, median lead time of longitudinal detectable MRD = 3.4 (m)	[53]
	Early-stage	181	PROPHET	LOD for PROPHET: 0.004%, sensitivity at baseline with ctDNA: 45%, median lead time of ctDNA detection over RI progression = 299 days	[54]
BRCA	Stage I–III	49	Signatera™	Sensitivity and specificity for recurrence: 89% and 100%, median lead time of ctDNA detection over RI progression = 8.9 (m), post-operative HR for RFS = 11.8	[13]
	Stage II–III	84	Signatera™	All pCR patients were ctDNA (−) after NAC, post-NAC HR for RFS in non-pCR patients = 10.4, non-pCR/ctDNA (−) was similar to pCR (HR = 1.4)	[19]
	Early-stage	55	dPCR	Post-operative HR for RFS = 25.1, serial HR for RFS = 12.0, median lead time of ctDNA detection over RI progression = 7.9 (m)	[64]
	Early-stage	170	dPCR	ctDNA (+) during follow-up: HR for relapse = 25.2, ctDNA (+) at diagnosis: HR for RFS = 5.8, median lead time of ctDNA (+) over clinical progression = 10.7 (m)	[65]
	Stage I–III	33	TARDIS	Sensitivity of TARDIS: 91% at AF of 0.003 and 53% at AF of 0.0003, median AFs: 0.003% in pCR patients and 0.017% in patients with residual disease	[66]
	High-risk stage II–III	83	RaDaR™	7.2% of patients developed recurrence, all of whom were ctDNA (+) before clinical recurrence with a median ctDNA lead time of 12.4 months	[67]
GU	UC (high-risk^c^)	581	Signatera™	ctDNA (+) treated with atezolizumab showed improved DFS (HR = 0.58), whereas no difference in DFS was observed between treatment arms for ctDNA (−)	[69]
	UC, localized	43	Oncomine Pan-Cancer Assay	Pre-operative ctDNA fraction > 2% was a significantly poor risk factor for RFS, post-operative ctDNA (+) was significantly associated with worse RFS	[70]
	BC, locally advanced	68	Signatera™	Pre-treatment HR for RFS = 29.1, ctDNA analysis identified all patients with metastatic relapse during disease monitoring (100% sensitivity, 98% specificity)	[71]
Others	EC, stage IA–IIIB	45	CAPP-Seq (607 genes)	Post-CRT HR for progression, distant metastases, and DSS = 18.7, 32.1, and 23.1, mean lead time of ctDNA detection over RI progression = 2.8 (m)	[72]
	HNSCC, locally advanced	20	Custom panel (127 genes)	ctDNA detectability: 85%, significant correlation between ctDNA and tumor volume, negative correlation between tumor allele fraction and treatment	[73]
	HNSCC, stage III–IVb	17	RaDaR™	Baseline ctDNA detectability: 100%, range of lead times for ctDNA detection prior to clinical recurrence: 108–253 days	[74]
	HCC, stage ≤ intermediate	96	Custom panel (1021 genes)	Recurrence rate in ctDNA (+) versus (−): 60.9% versus 27.8%, post-operative HR for DFS and OS = 6.074 and 4.829	[75]
	HCC, stage I–II	41	AVENIO	Pre-operative ctDNA (+): 63.4%, post-operative ctDNA (+): 46%, association of ctDNA positivity at two time points with (RFS): significantly shorter	[76]
	MM, stage III	99	ddPCR	Relapse in ctDNA (+) patients at baseline: 90%, relapse rate in post-operative ctDNA (+) patients: 100%, baseline and post-operative HR for RFS = 2.9 and 10	[77]

ctDNA, circulating tumor DNA; MRD, molecular residual disease; CRC, colorectal cancer; NSCLC, non-small cell lung cancer; BRCA, breast cancer; GC, gastric cancer; EC, esophageal cancer; PC, pancreatic cancer; GU, genitourinary cancer; UC, urothelial cancer; BC, bladder cancer; HNSCC, head and neck squamous cell cancer; HCC, hepatocellular cancer; MM, malignant melanoma; Safe-SeqS, safe-sequencing system; ddPCR, digital droplet polymerase chain reaction; CAPP-seq, cancer personalized profiling by deep sequencing; cSMART, circulating single-molecule amplification and resequencing technology; PROPHET, personalized tumor-informed technology; dPCR, digital polymerase chain reaction; TARDIS, targeted digital sequencing; CTx, chemotherapy; RFS, recurrence-free survival; ACT, adjuvant chemotherapy; DFS, disease-free survival; RI, radiographic imaging; pCR, pathological complete response; DSS, disease-specific survival, OS, overall survival; m, months; NAC, neoadjuvant chemotherapy; AF, allele frequency

^a^Number of patients evaluated for MRD by ctDNA

^b^Post-operative or landmark timepoints dependent on each study

^c^pT3–T4a or N + for patients that were not treated by neoadjuvant chemotherapy, or pT2–T4a or N + for patients treated by neoadjuvant chemotherapy

### Gastrointestinal cancer

#### Colorectal cancer

Colorectal cancer has a high degree of ctDNA shedding, making colorectal cancer a prime candidate for MRD evaluation via ctDNA with many clinical outcome reports supporting its utility [30]. Many studies have consistently shown that ctDNA after definitive therapy with curative intent, including surgery or its combination with chemotherapy, effectively predicts the relapse risk with relatively high sensitivities and specificities, often preceding clinical or radiological recurrence.

By applying a tumor-informed Safe-SeqS platform-based ctDNA assay to 230 and 96 patients with stage II and III colorectal cancer after surgery, respectively, Tie et al. reported that ctDNA positivity was an independent poor prognostic factor, and post-chemotherapy ctDNA analysis may define a patient subset that remains at high risk of recurrence despite completing standard adjuvant treatment [31, 32]. Moreover, Parikh et al. applied a plasma-only MRD assay, Guardant Reveal™ (Guardnant Health, Inc.), to 103 patients with stage I–IV colorectal cancer, who underwent curative-intent surgery, showing 55.6% sensitivity and 100% specificity for recurrence [33]. Similarly, various studies have reported the performance of MRD monitoring via ctDNA using both tumor-informed and -naïve assays for colorectal cancer [8, 34, 35].

The value of ctDNA-based MRD detection has recently been validated by large prospective trials. The DYNAMIC trial included 455 patients with stage II colorectal cancer, who were randomly assigned to have treatment decisions guided by ctDNA results or standard clinicopathological features. The ctDNA-guided management was found to effectively reduce the use of adjuvant chemotherapy while maintaining RFS compared with the standard-of-care, suggesting its viability as a treatment strategy [36].

In the GALAXY study, pre- and post-operative ctDNAs were analyzed in 1039 patients with stage II–IV resectable colorectal cancer. With a median follow-up of 16.74 months, post-surgical ctDNA positivity was significantly associated with an increased recurrence risk (HR 10.0, *P* < 0.0001), especially in patients with stage II or III colorectal cancer (HR 10.82, *P* < 0.001). Post-operative ctDNA positivity also identified stage II or III colorectal cancer patients who benefitted from adjuvant chemotherapy (HR 6.59, *P* < 0.0001), supporting ctDNA testing to identify patients at an increased recurrence risk who could benefit from adjuvant chemotherapy [14]. These findings herald a new era in personalized medicine for colorectal cancer, underscoring the critical role of quantifying ctDNA levels before and after surgery to customize therapeutic interventions aligned with the distinct recurrence risk of each patient.

#### Gastric cancer

In terms of gastric cancer and esophagogastric cancer, several studies focusing on advanced or metastatic tumors have underscored the prognostic significance of ctDNA levels [37–39]. Moreover, the utility of ctDNA to evaluate MRD after definitive therapy has been gradually reported in recent years. In a study of 46 patients with stage I–III gastric cancer, ctDNA prior to treatment was detected in 45% of patients using a tumor-informed assay targeted sequencing panel covering 1021 genes. All patients positive for ctDNA after curative surgery experienced recurrence. ctDNA positivity at any time during longitudinal post-operative follow-up was associated with poor DFS (HR = 14.78) and preceded radiographical recurrence by a median of 6 months [40]. In the CRITICS trial, a phase III randomized controlled study of perioperative treatment of patients with operable gastric cancer, 50 patients were analyzed using VariantDx (Personal Genome Diagnostics), identifying ctDNA alterations through ultrasensitive targeted sequencing analyses of matched cfDNA and white blood cells from each patient [41]. It demonstrated that pre-operative ctDNA is a biomarker for the pathological response, and ctDNA positivity after surgery indicated significantly short RFS (HR = 21.8) [42]. In 125 patients analyzed at any time point post-operatively regardless of adjuvant treatment using Signatera™, Huffman et al. reported that the recurrence rate was 88.2% among ctDNA-positive patients compared with 5.5% among ctDNA-negative patients, exhibiting a marked reduction in RFS (HR = 23.6) [43].

#### Pancreatic cancer

Patients with pancreatic cancer often have very low levels of ctDNA, necessitating ultrasensitive and reproducible approaches for clinical testing [30]. Sausen et al. reported that patients positive for ctDNA at various time points after surgery were more likely to relapse than those negative for ctDNA with recurrence detected by ctDNA 6.5 months earlier than CT imaging [44]. Groot et al.’s study investigating a *KRAS* ctDNA assay of 59 pancreatic cancer patients demonstrated that ctDNA positivity post-surgery was associated with an elevated risk of recurrence, making it a reliable predictor of clinical recurrence and survival outcomes [20]. Furthermore, several studies of resectable or borderline resectable pancreatic cancer patients have revealed that ctDNA-positive post-operatively was a significant poor prognostic factor [45–47].

#### Lung cancer

In the field of non-metastatic lung cancer treatment, primary surgical resection, radiotherapy, and comprehensive chemotherapy have shown efficacy to cure patients. However, imaging tests often detect recurrent or progressive lesions only after a significant increase in systemic tumor burden. Therefore, MRD detection following radical resection of lung cancer is gaining attention because of its potential to identify patients at risk of recurrence and to enable personalized adjuvant therapy before tumor progression. Some studies have shown that ctDNA analysis before or after surgery effectively predicts the relapse risk.

The TRACERx study marked a significant advancement by demonstrating the clinical utility of ctDNA for MRD detection in early-stage, non-small cell lung cancer (NSCLC) patients after curative treatment. Analysis using Signatera™ showed high sensitivity to detect ctDNA prior to clinical relapse with a median lead time of 70 days for ctDNA detection before radiographic confirmation of relapse in patients with NSCLC [18, 48]. However, lung adenocarcinoma was significantly less necrotic than lung squamous cell carcinoma with only 19.0% of lung adenocarcinoma cases positive for ctDNA (stage I, 5/39; stage II, 2/9; stage III, 4/10). An updated analysis using a patient-specific 50-variant anchored-multiplex PCR enrichment panel indicated an 82.2% relapse detection rate with a median ctDNA lead time of 151 days [49]. Nevertheless, among lung adenocarcinoma patients, only 41.9% were positive for ctDNA with lower positivity at earlier stages (stage I, 5/37; stage II, 12/27; stage III, 22/29). Further research employing a range of assays, including cancer personalized profiling by deep sequencing (CAPP-seq), the RaDaR™ assay (Inivata, Inc.), and personalized tumor-informed technology using deep sequencing of 50 patient-specific variants, has also suggested that post-operative plasma samples can be used to highly predict relapse and the utility of adjuvant therapy in NSCLC patients [50–56].

The correlation of ctDNA to the treatment response and prognostic significance in the context of neoadjuvant therapy or definitive radiation therapy have also been explored in NSCLC patients. Yue et al. reported a robust correlation between ctDNA dynamics during neoadjuvant therapy and the pathological response with pre- and post-surgery ctDNA levels associated with low RFS (HR = 7.41 and 5.37, respectively) [57]. Another study showed that ctDNA levels following neoadjuvant treatment were significantly linked to OS and surpassed radiological evaluations for survival prediction [58]. Additionally, Pan et al. observed dynamic ctDNA changes during chemoradiation therapy of patients with localized advanced NSCLC, identifying the positive prognostic and predictive value of early undetectable ctDNA [59]. These studies underscore ctDNA’s potential as a biomarker to gauge the effectiveness of neoadjuvant or definitive chemoradiation therapy and its prognostic relevance for NSCLC patients.

In future studies, correlative data of ctDNA clearance and MRD on outcome in practice-changing trials, such as IMpower010, PEARLS, and ADAURA, may further clarify the utility of ctDNA as a guide for neoadjuvant and adjuvant strategies [60–62]. These clinical trials validate a range of novel adjuvant treatments for MRD-positive patients with NSCLC. Presently, a prospective, multicenter study is underway, aimed at validating the hypothesis that no adjuvant therapy is necessary for patients who exhibit consistently undetectable MRD [63].

#### Breast cancer

In breast cancer, multiple studies have shown that MRD detection is strongly associated with disease recurrence with many months as the lead time prior to clinical evidence of recurrence. In 2015, Garcia-Murillas et al. reported a pivotal study including a cohort of 55 patients with high-risk, early-stage breast cancer treated by neoadjuvant chemotherapy [64]. They developed personalized tumor-specific digital-droplet PCR assays based on somatic mutations in primary tumors. They successfully predicted early relapse by tracking somatic mutations with a hazard ratio of 25.1 and lead time of 7.9 months. Another study showed that ctDNA was highly predictive of distant extracranial metastatic relapse across early-stage breast cancer subtypes with a median lead time of 10.7 months [65]. Further research on 49 high-risk stage I–III breast cancer patients showed that serial plasma ctDNA analysis using Signatera™ had 88.9% sensitivity and 100% specificity for relapse prediction with up to 2 years as the lead time [13]. McDonald et al. developed TARDIS (TARgeted DIgital Sequencing), demonstrating exceptional accuracy to identify the molecular response and residual disease in stage I–III breast cancer patients, enhancing the reliability and sensitivity of ctDNA MRD detection [66]. Lipsyc-Sharf et al. used the RaDaR™ assay and found that ctDNA effectively predicted recurrence more than 1 year before clinical recurrence, indicating the potential of ctDNA analysis to indicate early intervention in the late adjuvant setting of breast cancer [67].

The I-SPY 2 trial, a noteworthy neoadjuvant adaptive clinical trial designed to improve outcomes of high-risk breast cancer, indicated that serial ctDNA testing predicts a pathological complete response and metastatic recurrence risk in high-risk, early breast cancer patients treated by neoadjuvant chemotherapy [19, 68]. Nonetheless, Magbanua et al. reported the challenges of ctDNA detection due to subtypes among which ctDNA positivity was significantly lower in the hormone receptor-positive/human epidermal growth factor receptor 2 (HER2)-negative subtype (48.2%, 14/29) compared with HER2-positive (84.2%, 16/19) and triple-negative (86.1%, 31/36) subtypes.

Multiple MRD-guided clinical trials of adjuvant therapy with several classes of drug are currently underway. The LEADER trial (NCT03285412), DARE study (NCT04567420), and Trak-ER (NCT04985266) are evaluating the utility of *CDK4/6* inhibitors. The ZEST trial (NCT04915755) and c-TRAK-TN trial (NCT03145961) are evaluating the efficacy of adjuvant *PARP* inhibitors and pembrolizumab, respectively. The ASPRIA study (NCT04434040) is assessing the combination of atezolizumab and the antibody–drug conjugate sacituzumab govitecan. The PERSEVERE trial (NCT04849364) is a basket trial using ctDNA to guide post-neoadjuvant therapy, including talazoparib, atezolizumab, and inavolisib.

#### Genitourinary cancer

In 581 urothelial cancer (UC) patients who had undergone surgery and were evaluable for ctDNA from the IMvigor010 trial, a randomized phase III adjuvant study comparing atezolizumab to observation after surgical resection for operable UC, ctDNA testing at therapy initiation identified 37% of patients as positive for ctDNA, correlating to a poor prognosis (HR = 6.3). CtDNA-positive patients exhibited improved DFS (HR = 0.58) and OS (HR = 0.59) with no significant survival difference in ctDNA-negative patients between treatment arms. The ctDNA clearance rate at week 6 was higher in the atezolizumab arm (18%) than in the observation arm (4%) [69]. Nakano et al. reported that pre-operative ctDNA in 43 patients with localized UC was an independent risk factor for poor RFS, and early post-operative ctDNA positivity was significantly associated with poor RFS [70]. In terms of bladder cancer, serial ctDNA analysis of 68 patients who underwent neoadjuvant chemotherapy and surgery showed 100% sensitivity and 98% specificity for relapse detection with a median lead time of 96 days. Positive ctDNA indicated poor DFS and OS, and was a strong predictor of RFS post-cystectomy [71].

#### Other solid tumors

In terms of esophageal cancer, a study of 45 patients showed that ctDNA following chemo-radiotherapy markedly elevated the risk of disease progression and mortality. ctDNA detection also predicted relapse approximately 2.8 months prior to radiographic evidence with 71.4% sensitivity and 100% specificity [72].

In the context of head and neck squamous cell carcinoma (HNSCC), Hilke et al. evaluated 20 patients with locally advanced HNSCC treated by definitive chemoradiation therapy and suggested that ctDNA is a surrogate marker of disease burden, which markedly correlated to the tumor volume prior to the treatment [73]. Flach et al. investigated ctDNA in HNSCC patients who received primary surgical treatment with curative intent. Among 17 patients analyzed, all patients demonstrated ctDNA positivity in baseline samples collected prior to surgery. ctDNA successfully predicted clinical recurrence in all relevant patients with lead times ranging from 108 to 253 days [74].

In terms of liver cancer, Ye et al. assessed 96 patients with liver cancer and demonstrated that post-operative ctDNA was an independent prognostic predictor of DFS (HR = 6.07) and OS (HR = 4.83) [75]. Another investigation by Zhu et al. of 41 patients with hepatocellular carcinoma showed that the detection rate of ctDNA was 63.4% pre-operatively, which was reduced to 46% post-operatively. Pre-operative ctDNA positivity correlated to shorter RFS [76].

Regarding malignant melanoma, research on 99 patients with resected stage III melanoma indicated that ctDNA positivity after surgery served as a robust indicator of recurrence (HR = 10) and distant metastasis-free survival (HR = 11) [77].

### Challenges of MRD assessment by ctDNA in solid tumor patients

Although extensive innovations have improved ctDNA analysis, technical and biological factors that generate false-negative and false-positive results remain [78]. Variables, such as the total volume of plasma derived from whole blood, duration of sample storage, the procedures involved in specimen collection, transportation logistics, and processing protocols for blood specimens, collectively exert discernible effects on ctDNA analysis [79]. Furthermore, lifestyle behaviors, encompassing smoking, alcohol consumption, and physical exercise, in conjunction with physiological determinants, including inflammation, anemia, cardiovascular ailments, metabolic syndrome, autoimmune disorders, and even pregnancy, are considered to exert potentially corrupting effects on ctDNA measurements [80]. The precise mechanisms through which these multifaceted factors modulate the quality of blood specimens and analytical results represent a rich area for prospective investigation.

It has been posited that the tumor’s clinical status influences ctDNA. Advanced solid tumors typically exhibit elevated ctDNA release that is readily detectable by analytical methods. However, as observed in lung adenocarcinoma and breast cancer negative for HER2 and positive for hormone receptor, ctDNA levels are low and may be below the limit of detection by some MRD assays [18, 19]. Consequently, there is an urgent clinical need for enhanced assays that demonstrate improved sensitivity and analytical performance. Additionally, primary brain tumors and brain metastasis from solid tumors produce limited ctDNA, primarily owing to the impermeability of the blood–brain barrier [81]. Even in advanced colorectal cancer, which displays relatively high ctDNA release, various factors, such as post-primary tumor resection, oligometastatic disease, absence of liver metastasis, solely peritoneal dissemination or lung metastasis, diminished blood cell counts, low levels of tumor markers, and elevated albumin levels, have been proposed to attenuate ctDNA release rates [21, 22, 35, 82]. Notably, we reported diminished concordance between blood-based ctDNA and tissue-based mutational profiles in patients characterized by a limited tumor volume or only lung metastases [21, 22].

It is also important to note that hematopoietic stem cells often acquire somatic mutations with age, which is termed CHIP [23, 24]. This leads to the emergence of distinct cellular subclones in the hematopoietic system that are distinguished by their own driver mutations. These subclones can be disproportionately represented in the mature blood cell population [83], and may be linked to hematological disorders. However, CHIP is usually observed in older individuals who do not display overt hematological conditions [84–86]. Recent advancements in highly sensitive sequencing techniques have indicated that CHIP mutations are more prevalent than previously thought, being detected in up to 92–95% of patients, often at low allelic fractions [87]. This prevalence indicates a significant challenge to increase ctDNA detection specificity, because CHIP can lead to false positives, particularly when ctDNA is present at low levels, as observed in MRD scenarios [88]. To enhance ctDNA specificity, it might be beneficial to focus on clonal mutations, thereby avoiding CHIP variants with low allele fractions. Pairing ctDNA assays with sequencing of peripheral blood mononuclear cells may also improve specificity by filtering out the variants found in both [50]. Beyond CHIP, ctDNA analysis can be confounded by other forms of somatic mosaicism. These may arise from DNA replication errors or environmental factors causing genetic changes, such as losses, deletions, or duplications, which do not necessarily indicate malignancy [89]. Therefore, refining ctDNA detection methods to differentiate such somatic mosaicism by sequencing both peripheral blood mononuclear cells and plasma from healthy donors to filter the cell-free component is vital [30].

Consequently, the complexity of factors influencing ctDNA detection underscores the need for more sensitive and specific methodologies.

### Trajectory of the development of MRD detection by ctDNA in patients with hematological malignancies

The evolution of MRD detection in patients with hematological malignancies, which includes flow cytometry and PCR-based assays, has progressed differently from that in patients with solid tumors. MRD negativity is widely recognized as correlating to a good prognosis, particularly in patients with acute myeloid leukemia, acute lymphoblastic leukemia, and other hematological cancers [90–93]. MRD analysis, which has been centered on bone marrow samples, is increasingly incorporating ctDNA, especially for lymphoma patients, because of less invasive assessment. Standardization of MRD detection through immunoglobulin heavy chain/T-cell receptor (IgH/TCR) clonality sequencing with advancements, such as ClonoSEQ (Adaptive Biotechnologies), CAPP-Seq, and phased variant enrichment and detection sequencing (PhasED-seq) (Foresight Diagnostics), is underway [94]. However, in patients with myeloid malignancies, especially acute myeloid leukemia, the role of NGS in MRD detection remains to be firmly established [95].

The prognostic significance of MRD in patients with various hematological malignancies is being increasingly recognized, and its status is used in clinical trials to guide treatment strategies [90–96]. Despite its growing importance, MRD data are often excluded from U.S. prescribing information because of analytical, validation, and trial design complexities [97]. However, incorporation of MRD as an endpoint in phase II clinical trials for hematological malignancies is gaining momentum and consideration [98].

MRD assays for patients with hematological malignancies vary by the disease. For *BCR-ABL-*positive leukemia patients, RT-PCR targeting *BCR-ABL* is used, whereas multiparametric flow cytometry (mpFC) and allele-specific oligonucleotide real-time quantitative PCR are employed for patients with B and T cell malignancies [99]. However, mpFC faces challenges in standardization and sensitivity. In the U.S., ClonoSEQ is employed for MRD assessment of several B and T cell malignancies [100]. For patients with acute myeloid leukemia and myelodysplastic syndromes, MRD assessment primarily employs mpFC of bone marrow samples. In specific genetic mutation cases of acute myeloid leukemia patients, MRD is evaluated by PCR or NGS [92, 101]. However, the complexity of relapsing clones and the distinction between mutations suitable for MRD evaluation and those associated with clonal hematopoiesis present challenges [102]. For leukemia patients with bone marrow fibrosis or primary myelofibrosis, MRD assessment using bone marrow biopsy specimens or ctDNA has become necessary.

NGS-based MRD testing of acute myeloid leukemia patients is promising, but it is limited by high sequencing errors, a long turnaround time, and high costs. The European LeukemiaNet 2022 guidelines recommend error-corrected sequencing for NGS assays, especially single-nucleotide variant detection in MRD patients [103]. Innovative methods using ctDNA for disease monitoring are being developed and may replace invasive bone marrow assessments [104, 105]. MRD detection by ctDNA has challenges, but it is advancing with studies focusing on target cell enrichment techniques to enhance the clinical significance and validity of these measurements [106, 107].

### Methodology of ctDNA detection by tumor WGS analyses

**Fig. 1:**
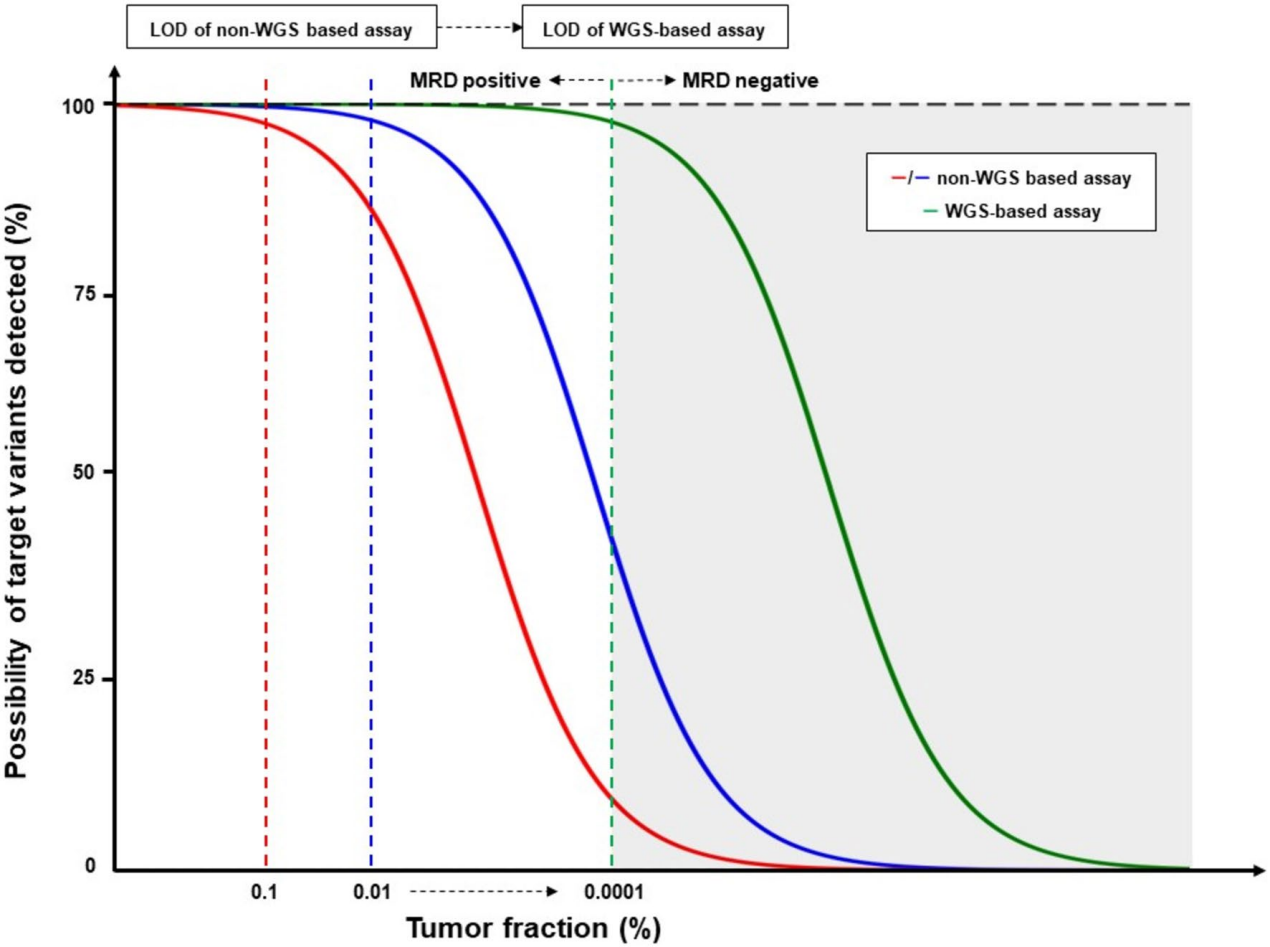
Improvement in performance of MRD detection. The figure illustrates the hypothetical improvement in performance of ctDNA-based MRD assays. When the limit of detection for the conventional non-WGS-based assay hovers around 0.1–0.01, in contrast, the limit of detection for the WGS-based assay is approximately 0.0001, there is a marked escalation in the possibility of target variants detected, enabling identification of MRD via ctDNA that has been undetectable by non-WGS-based assays

To overcome technical constraints, investigators often increase the sequencing depth to enhance detection of ctDNA mutations. However, the improvement is often limited because a typical blood draw contains a limited number of cfDNA molecules [26]. Conversely, using WGS to identify a comprehensive pool of targetable variants and then tracking a large number of them in plasma (exceeding 1000 variants), while potentially sequencing deeply enough to detect all available molecules at each targeted site, yields higher statistical power to detect residual ctDNA. Consequently, we have reviewed several reports that employed WGS to develop high-performance MRD assays (Table 2). These assays surpass the limit of detection thresholds of non-WGS-based methods, enabling identification of ctDNA that has been undetectable (Fig. 1).

**Table 2:** Summary of the platforms for ctDNA-based MRD detection by whole genome sequencing

Assay	Company	Methods		Reported/expected LOD
		Baseline	MRD detection via ctDNA	
NEXT Personal	Personalis	Tumor-normal DNA WGS	Hybrid capture-based NGS	TF = 0.0001–0.0003%
MRDetect	Veracyte	Tumor-germline DNA WGS	WGS	TF = 0.001%
PhasED-Seq	Foresight Diagnostics	Paired tumor-normal WGS	Hybrid capture-based NGS	TF < 0.0001%
MAESTRO	–	Tumor-normal DNA WGS	Hybrid capture-based NGS	Median TF = 0.00037% [range 7.9 × 10^–7^–0.49]
				Median TF = 0.00011% [range 7.8 × 10^–7^–0.13]
Precise MRD	Myriad genetics	Paired tumor-normal WGS	Hybrid capture-based NGS	Expected LOD95 TF = 0.0001%–0.001%

WGS, whole genome sequencing; MRD, molecular residual disease; LOD, limit of detection (ppm); MAESTRO, minor allele-enriched sequencing through recognition oligonucleotides; WES, whole exome sequencing; PCR, polymerase chain reaction; NGS, next-generation sequencing; VAF, variant allele frequency; TF, tumor fraction

#### NeXT personal (Personalis)

This is an ultrasensitive, WGS-based MRD detection platform with > 99.95% specificity in detecting low ctDNA levels in lung cancer patients [108, 109]. In a study involving 171 early-stage lung cancer patients, it effectively identified ctDNA in 81% of adenocarcinoma cases across various stages and in all non-adenocarcinoma patients. High pre-operative ctDNA levels were linked to poor survival outcomes. This approach may predict prognosis and guide therapy of lung cancer patients, especially early-stage patients at high risk of relapse.

#### MRDetect (Veracyte)

MRDetect is a WGS‑based cfDNA assay for MRD detection. All somatic alterations and copy number alterations identified by WGS are used to inform each personalized ctDNA assay [110, 111]. To integrate the genomic signature with machine‑learning artificial intelligence‑based error suppression models, this assay requires a low amount of input plasma and exhibits an limit of detection of 0.001% tumor fraction at a genome‑wide sequencing depth of 35 ×. Performance of the test depends on the tumor mutation burden (TMB). Tumors with a high TMB will have a better limit of detection than those with a low TMB. MRDetect efficacy was validated by simulations and clinical studies, showing that positive MRD detection is associated with shorter disease-free survival in colorectal cancer and lung adenocarcinoma patients.

#### PhasED-seq (Foresight diagnostics)

PhasED‑seq is a hybrid capture‑based sequencing assay designed to detect phased variants, significantly enhancing sensitivity compared with traditional single nucleotide variant-based MRD assays [94]. This method has achieved remarkable improvement in sensitivity with a limit of detection below 0.0001% tumor fraction for some tumor types. The number of phased variants, and thus, the limit of detection of PhasED-seq, varies across cancers and depends on the TMB of a tumor. Low TMB tumors may only have some phased variants, which would bring the limit of detection of PhasED-seq in line with assays targeting unphased variants. Using WGS data from 2538 tumors, PhasED-seq identifies phased variants and their connections to mutational signatures. Its application extends beyond diffuse large B cell lymphoma to various solid tumors.

#### Minor allele-enriched sequencing through recognition oligonucleotides (MAESTRO)

MAESTRO is a novel method that enhances the ability to track a large number of low-frequency mutations, overcoming the limitations of high-depth sequencing required for such tasks [112–114]. This technique integrates massively parallel mutation enrichment with duplex sequencing, allowing for tracking of up to 10,000 low-frequency mutations using up to 100 times fewer reads per locus compared with conventional hybrid-capture duplex sequencing. MAESTRO has been successfully applied in various contexts, including testing for chimerism in human cell lines, validating mutations in breast tumor samples, and monitoring MRD in patients.

#### Precise MRD (Myriad genetics)

Precise MRD identifies somatic variants by tumor-normal WGS and uses machine learning to select an optimized set of hundreds to thousands of tumor-specific variants to probe via hybridization capture of plasma-derived, unique molecular identifier-barcoded cfDNA. Spanning stage I–III cancers in nine indications, 80% of samples had ≥ 1000 targetable high-confidence variants, and 97% of samples had > 300 targetable variants [115]. While maintaining specificity of > 99%, sensitivity was 95% down to a tumor fraction of 0.002% with limits of detection in the parts-per-million range achievable by marginally lower specificity.

### Next SCRUM-MONSTAR-SCREEN project: MONSTAR-SCREEN-3

**Fig. 2:**
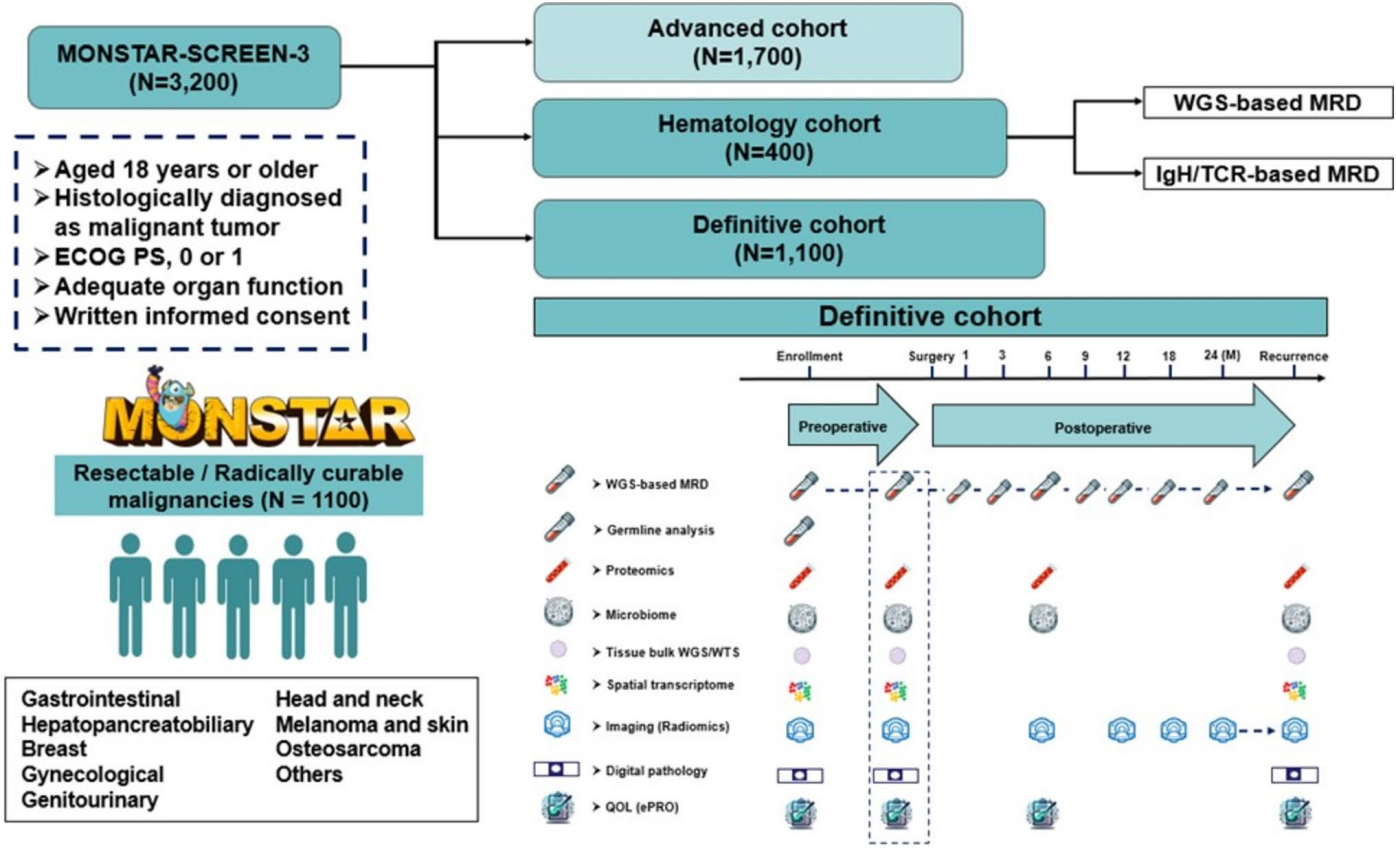
Pan-cancer WGS-based MRD monitoring project. The MONSTAR-SCREEN-3 trial will include 3200 patients: 1700 patients for an advanced cohort, 1100 patients for a definitive cohort, and 400 patients for a hematology cohort. For the definitive cohort, multiomics analyses will primarily concentrate on molecular residual disease (MRD) detection in all solid tumor patients, particularly through whole genome sequencing (WGS)-based MRD analysis, along with WGS, whole transcriptome sequencing, and spatial transcriptomics of tissues, plasma proteomics, microbiome analysis, and germline profiling. Additionally, radiological and pathological images will be digitized for inclusion in our database of all cohorts, and electronic patient-reported outcomes for quality-of-life evaluations will also be integral components of the definitive cohort. In the hematology cohort, 400 patients will be divided into two groups: 200 with leukemia and 200 with non-leukemia. MRD analysis of leukemia patients will involve WGS-based MRD assessment, whereas patients with non-leukemia will undergo immunoglobulin heavy chain/T-cell receptor-based MRD detection methodology

We anticipate finalizing the enrollment of 2750 participants by March 2024 in our ongoing clinical study, MONSTAR-SCREEN-2. The MONSTAR-SCREEN-3 trial is scheduled to commence in April 2024, aiming to include 3200 patients. This trial includes three distinct subgroups: an advanced cohort for treating advanced solid tumors with systemic pharmacotherapy, a definitive cohort targeting radically resectable solid tumors with curative treatment modalities, and a hematology cohort focusing on hematological malignancies (Fig. 2).

In the advanced cohort, comprehensive molecular characterization employing multiomics techniques will be applied. This includes whole exome sequencing (WES) and whole transcriptome sequencing (WTS) of circulating tumor DNA/RNA, bulk WES/WTS and spatial transcriptomic sequencing of tissue specimens, proteomics profiling of plasma samples, germline analysis of buffy coats and normal tissues, and fecal microbiome analyses before treatment initiation and post-disease progression. For the definitive cohort, multiomics analyses will primarily concentrate on MRD detection in all solid tumor patients, particularly through WGS-based MRD analysis, along with WGS, WTS, and spatial transcriptomics of tissues, plasma proteomics, microbiome analysis, and germline profiling. The hematology cohort will consist of patients with hematological malignancies, including leukemias and myeloid malignancies, such as acute myeloid leukemia, myelodysplastic syndromes, and myeloproliferative neoplasms, and non-leukemias and lymphoid malignancies, such as lymphomas and multiple myelomas. Multiomics analyses including MRD monitoring, WES, WTS, spatial transcriptomic assessments, plasma proteomics, microbiome analyses, and germline profiling will be conducted. Additionally, radiological and pathological images will be digitized for inclusion in our database of all cohorts, and electronic patient-reported outcomes for quality-of-life evaluations will also be integral components of the definitive cohort.

### Pan-cancer WGS-based MRD detection project

In the MONSTAR-SCREEN-3 initiative, definitive and hematology cohorts will be primarily analyzed by longitudinal surveillance of MRD using the ultrasensitive WGS-based assay Precise MRD in collaboration with Myriad Genetics. Considering its analytical performance, low sample input requirements, and applicability to pan-cancer MRD detection, Precise MRD has the potential to meaningfully showcase the prognostic and predictive value of MRD in various cancers.

For the definitive cohort, MRD evaluations will encompass both pre-treatment, pre-operative, and post-operative phases, particularly in scenarios involving pre-operative therapy. In the case of up-front surgery, MRD evaluations will be limited to pre- and post-operative timepoints. This cohort will also undergo sequential postoperative MRD evaluations at specified junctures: 1 month post-surgery, quarterly during the initial year, biannually in the subsequent year, and upon recurrence manifestation. The enrollment target includes 1100 patients with all solid tumors. The spectrum of solid tumors encompasses a wide array of malignancies, including colorectal cancer, gastric cancer, pancreatic cancer, esophageal cancer, biliary tract cancer, hepatocellular carcinoma, head and neck cancer, urothelial cancer, renal cell carcinoma, breast cancer, ovarian cancer, endometrial cancer, cervical cancer, malignant melanoma, small intestinal cancer, neuroendocrine neoplasm, anal canal cancer, appendiceal cancer, osteosarcoma, and others.

In the hematology cohort, 400 patients will be divided into two groups: 200 with leukemia and 200 with non-leukemia. MRD analysis of leukemia patients will involve WGS of bone marrow tissues and WGS-based MRD assessment, whereas patients under non-leukemia conditions will undergo MRD analysis using IgH/TCR-based MRD detection methodology.

The definitive cohort encompasses all solid tumor categories with each cancer type having a capped enrollment, generally not exceeding 100 patients. However, contingent upon scientific interest from academic or pharmaceutical entities, and upon successful feasibility demonstration and proof-of-concept for the MRD assay in specific cancer subgroups, these cohorts might be expanded. This approach has the potential to revolutionize the scope of WGS-based MRD projects and foster the development of MRD-guided therapeutic strategies for a broad spectrum of cancers.

## Conclusions and future perspectives

**Fig. 3:**
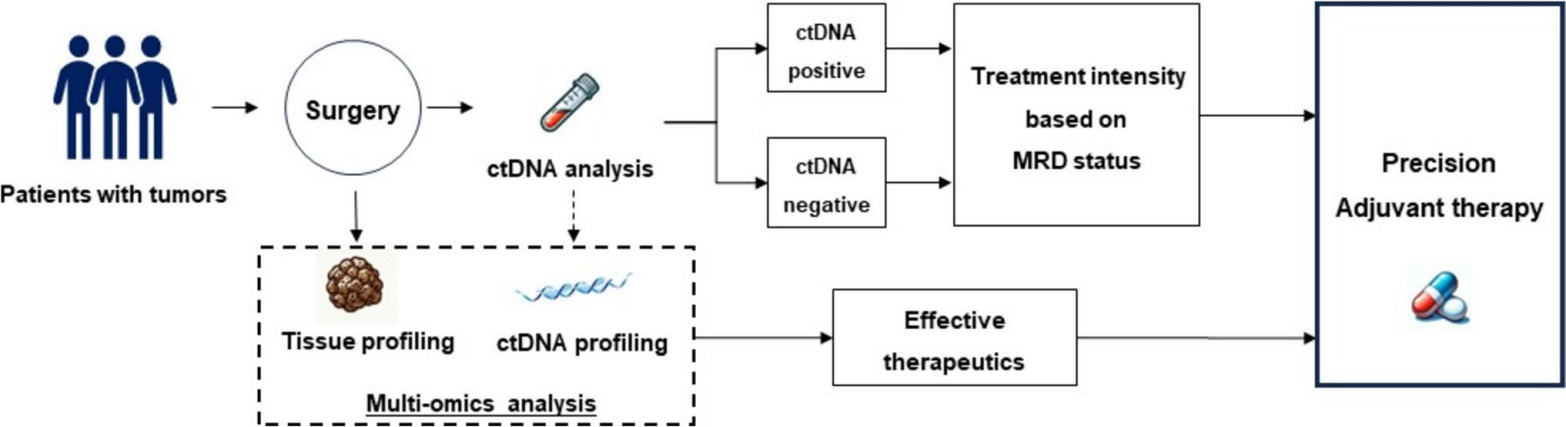
Perspective of precision medicine using ctDNA-based MRD detection. Post-surgical molecular residual disease (MRD) status (positive or negative), following curative-intent procedures, facilitates stratification of treatment intensity (either escalation or de-escalation). Moreover, comprehensive molecular profiling of surgical specimens, circulating tumor DNA, tumor microenvironment, and host immune responses, paves the way for the development of efficacious therapeutic interventions. By integrating these data, there exists the potential to craft precision, personalized adjuvant chemotherapy

Rapidly expanding evidence strongly supports the effectiveness of ctDNA-based MRD detection to predict tumor relapse of various cancer types, which is distinguished by its remarkable sensitivity and precision. Nonetheless, numerous challenges persist in assimilating this modality into standard clinical practice because its comprehensive clinical utility across diverse cancers is yet to be fully realized. To overcome these issues, we aim to implement the WGS-based MRD detection platform via ctDNA for all tumor types, including hematological malignancies, in our upcoming project. If the effectiveness of these MRD assays is verified either universally or for specific tumor types, clinical trials can be vigorously pursued to select treatment strategies based on the post-operative MRD status, which is similar to our ongoing VEGA and ALTAIR trials, or to develop more personalized therapies that integrate ctDNA analysis with extensive host and tumor multiomics (Fig. 3). In parallel with this endeavor, we are currently developing an MRD assay that incorporates a multiomics approach, including whole genome, transcriptome, proteome, metabolome, and microbiome analyses. For this purpose, we have initiated a collaboration with the Tohoku Medical Megabank Organization renowned for having one of the world’s largest biobanks of healthy individuals. Our goal is to create an innovative MRD assay that combines multiomics data from both cancer patients and healthy individuals using artificial intelligence.

In view of the current landscape, treatment strategies can be adaptively refined by ctDNA-based MRD detection and monitoring after surgery. Despite numerous technical challenges, ctDNA-based MRD assays are undoubtedly poised to become increasingly instrumental in the personalized post-operative management of patients with various tumor types in the foreseeable future.
